# Data collection for mobile crowdsensing in the presence of selfishness

**DOI:** 10.1186/s13638-016-0580-x

**Published:** 2016-03-15

**Authors:** Jieyan Liu, Lubomir Bic, Haigang Gong, Siyu Zhan

**Affiliations:** School of Computer Science and Engineering, University of Electronic Science and Technology of China, Chengdu, China; Donald Bren School of Information and Computer Sciences, University of California, Irvine, USA

**Keywords:** Selfishness, Routing, Data collection, Cooperative willingness

## Abstract

Mobile crowdsensing is an emerging approach to data collection by exploiting the sensing abilities offered by smart phones and users’ mobility. Data collection can be implemented by exploiting the forwarding opportunities given by the contacts between nodes. However, as cell phones are still resource constrained, most people are socially selfish so that they may not always cooperate with each other in data collection. In this paper, we propose a routing protocol, called Accept aNd Tolerate (ANT), which is tailored for data collection in a social environment with selfish individuals. ANT works by accepting and tolerating social selfishness as an unavoidable human characteristic. It makes relay selection based on nodes’ contacts and their willingness to cooperate. The cooperative willingness of selfish nodes is measured rationally according to the reciprocity relationship between nodes and their resource constraints. Through assessing the worthiness of carrying and forwarding a packet, ANT proposes a buffer management scheme and makes forwarding decisions. Simulations based on real traces show that ANT achieves better performance under resource-constrained circumstances than other comparable approaches.

## Introduction

Mobile crowdsensing is a novel approach that exploits the sensing capabilities offered by smart devices such as smart phones to sense and generate collective knowledge about a phenomenon or condition of interest [1]. These data may be measurement samples, text, and even photographs or video clips. Since it can utilize the mobility of users to solve large-scale mobile-sensing tasks, it has stimulated a number of attractive applications, such as urban WiFi characterization [2], traffic information mapping and parking space management [3], environmental monitoring, and social journalism.

**Fig. 1:**
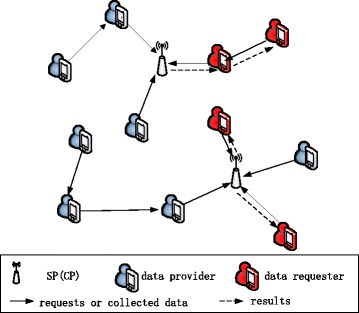
Overview of the mobile crowdsensing system

A typical mobile crowdsensing system is shown in Fig. 1. It involves three main actors: (1) the end users (data providers) who contribute the sensor data, (2) the service provider (SP, also viewed as the collection point (CP)) processing the collected data to generate a service from them, and (3) the end users (data requesters) who request this service [4, 5]. Data collection (including request collection from the data requesters and data collection from the data providers) is an essential block to build mobile crowdsensing systems [6, 7]. Some current literature assumes that the end users use the cellular network resources for transferring data to the collection point as soon as these are generated by their devices sensors. However, this approach increases the communication cost and generates additional workload for the cellular network. This problem becomes worse when large amounts of data are generated (e.g., when the data type is quality photo) or when it takes place during the network busy hours. Opportunistic networking is viewed as a promising complement to cellular networks in different respects, e.g., for offloading delay-tolerant traffic load from them [8]. Thus, data collection in mobile crowdsensing can be implemented by using the contact opportunities among nodes when the application is delay tolerant, i.e., the mobile user sends data to another mobile user via Bluetooth when they encounter each other or via WiFi when they visit a collection point, as shown in Fig. 1.

Routing in such scenarios for data collection is analogous to the routing in delay-tolerant networks (DTNs) [9]. However, existing routing solutions [10–14] for the social environment of DTNs may not be applicable, as these implicitly assume that nodes are fully cooperative with each other in data relaying. In reality, as mobile phones are still resource constrained and are controlled by individuals, they may behave in a socially selfish manner and may not always cooperate in packet relaying. Similar to the behavior of human beings, nodes are usually cooperative based on the reciprocal social relationship. However, their cooperative willingness is affected by their resource status. When their resources are low, the probability of rejecting others’ requests is high. In this paper, we refer to the above feature as *selfishness*.

Although some studies [14–19] propose incentive schemes to stimulate selfish nodes to cooperate, they go into the extreme by attempting to completely eliminate nodes’ selfishness. To make routing protocols work well for data collection in a social environment, it is necessary to accept selfishness as an unavoidable feature and develop algorithms that can tolerate it, rather than trying to eliminate it. Some initial work has been conducted in [20–22]. However, these approaches neglect the effect of resource status on nodes’ willingness to cooperate.

In this paper, we propose a routing protocol, referred to as *A*ccept a*N*d *T*olerate (ANT) tailored for data collection for mobile crowdsensing in the presence of social selfishness. ANT works by accepting and tolerating social selfishness as an unavoidable feature. It evaluates nodes’ delivery abilities by combining the contact opportunities and their cooperative willingness. Through emulating the nature of people, ANT assesses the cooperative willingness of selfish nodes according to the reciprocal social relationship and their resource constraints. It also presents a scheme of measuring the degree to which a packet is worth carrying and employs that in buffer management and forwarding decisions. Extensive simulations based on the real traces show that ANT achieves good delivery performance with low transmission cost.

The remainder of this paper is structured as follows. Section 2 makes a brief overview of related work. In Section 3, we introduce the design of ANT in detail. Section 4 evaluates ANT through realistic experiments. Finally, Section 5 summarizes the paper.

## Related work

Data collection is an essential part of building an emerging people-centric sensing system [1, 4, 5]. Due to the intermittent connectivity caused by nodes’ mobility, the problem of routing the sensor data to the collection points is analogous to the routing problem in DTNs [9]. The prevalent solution for routing in the social environment of DTNs is to use the properties of human mobility and relationships for relay selection. Examples of this include Simbet [10], which exploits the “small-world” phenomenon of human society and employs “betweenness” centrality and social similarity to diffuse packets from sources to destinations; BubbleRap [11], which combines the knowledge of community structure with the centrality of each node to make a routing decision; PeopleRank [12] and Social-greedy [13], which also exploit several social dimensions to achieve efficient packet transmission. However, all of these solutions rely on the altruistic cooperation among nodes, which may not always be true in reality as nodes suffer from resource constraints and may behave selfishly.

Recent research [23, 24] has proven that the performance of data forwarding can be affected gravely when nodes behave selfishly. In view of that, some incentive approaches have been proposed to mitigate the impact of selfishness on the performance. These solutions can be classified into three main categories: reputation-based approaches [14, 15], credit-based approaches [16, 17], and game-based approaches [18, 19]. In reputation-based approaches, nodes collectively detect misbehaving members and propagate declarations of misbehavior throughout the network. Eventually, this propagation leads to other nodes’ avoidance of routes through selfish members. In credit-based approaches, nodes pay and get paid for providing service to other nodes. In game-based approaches, a game-theoretical model is developed to prove that the approach fosters cooperation among the nodes. The common objective of all these approaches is to encourage selfish nodes to cooperate; however, they all fall into the extreme by attempting to completely eliminate the selfish behavior of nodes.

As social selfishness is an inherent feature of most nodes, some recent researches have made efforts to design routing protocols that can accept social selfishness as an unavoidable fact and allow nodes to be socially selfish. Give2Get (G2G) [20] studies the egocentric behavior of nodes and develops the Give2Get epidemic and Give2Get delegation-forwarding algorithms, where nodes are selfish with outsiders and faithful with the nodes from the same community. Social Selfishness Aware Routing (SSAR) [21] quantifies nodes’ cooperative willingness based on the social relationship and evaluates the social relationship based on the contact frequency among nodes. However, this is not always accurate in reality. For instance, most people are willing to forward packets for those nodes that have often forwarded packets for them, even if there are no frequent contacts between them. Meantime, even when a close social tie exists, a node may refuse to help others when its resources are low. Although Hot-area-based Selfish Routing (HASR) [22] considers the impact of resource constraints, it lacks a refined evaluation of how the resource status affects the nodes’ cooperative willingness.

## Proposed ANT

### ANT overview

The working methodology of the proposed ANT is summarized below.

ANT forwards packets based on a delegation approach. A node will forward the packet only if it encounters the collection point or another node with better delivery ability. The delivery ability of a node is evaluated by combining its cooperative willingness with the contact history between the node and the collection point.

The cooperative willingness of a node is measured from two aspects: the reciprocity relationship between nodes and the resource constraints. This notion emulates the nature of people in that people tend to help those who reciprocate the help, but the level of kindness is also affected by their resource status.

To maximize the utilization of the limited buffer and the forwarding opportunities, ANT incorporates the type and *quality* of a packet to assess the degree to which a packet is worth carrying and forwarding. Based on that, it proposes a buffer management scheme and makes the forwarding decisions.

### Delivery probability

We use delivery probability to measure the possibility that a node can deliver packets to the collection point successfully. Most traditional approaches evaluate a node’s delivery ability only based on the contacts between the node and the collection point. However, it is worth noticing that the success of data delivery in a socially oriented environment does not depend only on the contacts, because nodes can be either cooperative or uncooperative in data relaying. If a node contacts the collection point frequently but it is reluctant to carry and forward packets for others, it is not a good candidate for a relay as the possibility that it drops the packet is high. In view of that, ANT evaluates the delivery ability of a node from two aspects. One is the list of contacts between the node and the collection point, and another is the cooperative willingness, which reflects how much a node is willing to carry and forward packets for the source node. These two aspects are independent; thus, the possibility that node *i* can deliver packet *m* to the collection point, denoted by *D*_*m*_(*i*), can be formulated as

1 $$ {D}_m(i)=C\left(i,\varDelta \right)\times {W}_m^{\mathrm{com}}(i) $$

where *Δ* denotes the collection point and *C*(*i*, *Δ*) is the possibility that node *i* can contact *Δ* during time interval *T* and ${W}_m^{\mathrm{com}}(i)$ is the comprehensive cooperative probability that node *i* is willing to carry and forward packet *m*. (The methodology of computing ${W}_m^{\mathrm{com}}(i)$ will be discussed in detail in Section 3.3.) *C*(*i*, *Δ*) is given by

2 $$ C\left(i,\varDelta \right)={\displaystyle {\sum}_{q=1}^K{t}_q}\left(i,\varDelta \right)/T $$

where *K = fT*, *f* is the contact frequency between node *i* and the collection point, and *t*_*q*_(*i*, *Δ*) is the *q*th contact duration. Node *i* updates *C*(*i*, *Δ*) when *T* expires.

3 $$ C\left(i,\varDelta \right)=C\left(i,\varDelta \right)^{\prime}\times \alpha +\left[C\left(i,\varDelta \right)\right]\times \left(1-\alpha \right) $$

where *C*(*i*, Δ)′ is the old contact probability before updating, [*C*(*i*, *Δ*)] is the contact probability obtained in the latest period based on Eq. (2), and 0 ≤ *α* ≤ 1 is a constant employed to keep partial memory of the historic status.

### Cooperative willingness

Cooperative willingness of a node reflects how much the node is willing to carry and forward packets for the source node. ANT uses the cooperative probability to measure the cooperative willingness of a node. Following the typical behavior of human beings, the cooperative willingness is often affected by two factors: the *reciprocity factor* and the *resource factor*. Suppose *s* is the source node of packet *m* and let ${W}_{\mathrm{rec}}^s(i)$ be the *reciprocity factor* of node *i*, i.e., the probability that node *i* is willing to carry and forward packets *m* for node *s* based on the historical contributions that node *i* and node *s* have made to each other in data relaying. Let *W*_res_(*i*) be the *resource factor*, i.e., the probability that node *i* is willing to carry and forward packets for others based on its resources status. (Details of how to compute the two factors will be illustrated in the next two sections.) The comprehensive cooperative probability that node *i* is willing to carry and forward packet *m* for node *s* is formulated as

4 $$ {W}_m^{\mathrm{com}}(i)=\left(1-\mu \right)\times {W}_{\mathrm{rec}}^s(i)+\mu \times {W}_{\mathrm{res}}(i) $$

where 0 ≤ *μ* ≤ 1 is a tunable parameter, which allows for the adjustment of the relative importance of the two factors. This means there is a trade-off between the two factors and it is adjusted dynamically based on the resources status. We can model the adaptive weight *μ* as a strictly monotonically decreasing function of *W*_res_(*i*). Since it is impossible to traverse all possible solutions for *μ*, we apply the linear and the logarithmic solutions that *μ* = 1 − *W*_res_(*i*) and *μ* = 1 − 1/(1 − ln *W*_res_(*i*)), respectively, in our work. Both solutions have the common objective to assign an increasing adaptive weight to the *resource factor* to ensure that nodes’ cooperative willingness depends more on the *resource factor* when the available resource becomes less, but it depends more on *reciprocity factor* when the resource is sufficient. Specifically, *μ* is close to 1 when *W*_res_(*i*) is close to 0, and *μ* is close to 0 when *W*_res_(*i*) is close to 1 and *μ* = 0 when *W*_res_(*i*) = 1. We apply these two solutions in the simulation and only present the results of the best solution, i.e., *μ* = 1 − *W*_res_(*i*), in Section 4.

#### Reciprocity factor

The *reciprocity factor* is based on nodes’ reciprocal relationship. Let *L*_*is*_ be the reciprocity level between node *i* and node *s*, which is given by

5 $$ {L}_{is}=\left\lceil \frac{N_{is}}{H}\right\rceil $$

where *N*_*is*_ is the number of packets that node *i* has relayed for node *s* and *H* is the range of each level. *L*_*is*_ and *L*_*si*_ may not be equal as *N*_*is*_ and *N*_*si*_ may not be the same. The *reciprocity factor* of node *i* is calculated as

6 $$ {W}_{\mathrm{rec}}^s(i)=\left\{\begin{array}{l}1\begin{array}{ccc}\hfill \hfill & \hfill \hfill & \hfill \mathrm{if}\hfill \end{array}\left({L}_{si}\ge {L}_{is}\right)\\ {}\begin{array}{cc}\hfill {L}_{si}/{L}_{is}\hfill & \hfill \mathrm{if}\hfill \end{array}\left({L}_{si}<{L}_{is}\right)\end{array}\right. $$

When *L*_*si*_ ≥ *L*_*is*_, node *i* is totally willing to forward packets for node *s* to express gratitude. However, when *L*_*si*_ < *L*_*is*_, ${W}_{\mathrm{rec}}^s(i)$ depends on the proportion of *L*_*si*_ to *L*_*is*_, that is, the smaller *L*_*si*_ is than *L*_*is*_, the lower the possibility that node *i* is willing to forward packets for node *s*.

To reduce the computation cost, node *i* does not have to compute *L*_*is*_ whenever *N*_*is*_ is updated. It only updates *L*_*is*_ when it has forwarded a certain number of packets (e.g., *H* packets) for node *s* since the last update. When the reciprocity level is updated, the exchange of the updates between nodes can be piggyback-transmitted on the regular beacon messages. Here we assume the beacon messages are exchanged faithfully, and we leave the security issues as our future work.

#### Resource factor

The *resource factor* is based on the nodes’ resource status. It is natural that one’s cooperative willingness will change with the change of its available resources. For example, one’s cooperative willingness will decline when the residual battery energy decreases. ANT uses a composite cooperative willingness function ƒ(*R*) to compute *W*_res_(*i*), where ƒ(*R*) is a combined function of all resource indicators. Specifically, considering *n* types of resources with associated cooperative willingness functions *R*_1_,…, *R*_*n*_, the problem can be reformulated as a multiple-criteria decision problem [25] with *n* goals:

7 $$ {W}_{\mathrm{res}}(i)=f(R)=f\left({R}_1,\dots, {R}_n\right) $$

The combined cooperative willingness function, using the weight method, can be defined as

8 $$ {W}_{\mathrm{res}}(i)={\displaystyle {\sum}_{z=1}^n{w}_z{R}_z} $$

where *w*_*z*_ is the significance weight reflecting the relative importance of the *z*th resource status on the cooperative willingness according to the desire of users, and ${\displaystyle {\sum}_{z=1}^n{w}_z=1}$. The overall cooperative willingness function ƒ(R) gives a measure of the probability that node *i* is willing to carry and forward packets for others based on its resource status. The solution for each cooperative willingness function *R*_*z*_ is that when the available amount of resource *z* is more than or equal to a predefined threshold, the cooperative willingness is 1. When it is less than the threshold, we use different levels to indicate the different amount of the resource and a reference level is associated with the threshold, and *R*_*z*_ is achieved based on the proportion of the current level against the reference level. For instance, suppose the threshold for the buffer space is 60 % of the buffer size, the range of each level is 10 % of the buffer size and the reference level is 6. When the available buffer space is 80 % of the buffer size, it is larger than the threshold and *R*_*z*_ = 1. When the available buffer space is 40 % of the buffer size, the current level is 4 and *R*_*z*_ = 0.67 (4/6).

### Buffer management

#### Packet quality

Each packet is associated with a *quality* to measure the degree to which the packet is worth carrying under a resource-constrained situation. For any packet *m* on node *i*, whether it is worth carrying is determined by several factors. One is the probability that node *i* can deliver packet *m* successfully. The higher *D*_*m*_(*i*) is, the more packet *m* is worth keeping. Another factor is the probability that packet *m* can be delivered successfully by other carriers, denoted by *D*_*m*_(*Ω*), where *Ω* is the set of other carriers known by node *i*. For node *i*, packet *m* is less worth keeping when *D*_*m*_(*Ω*) is high. *D*_*m*_(*Ω*) is given by

9 $$ {D}_m\left(\varOmega \right)=1-{\displaystyle {\prod}_{x\in \varOmega}\left(1-{D}_m(x)\right)} $$

When node *i* meets node *j*, it adds node *j* into *Ω* if it replicates packet *m* to node *j* or it exchanges Ω with node *j* if node *j* also carries packet *m*.

Apart from the above two factors, two other factors concerning the worthiness of carrying packet *m* are ${P}_m^{\mathrm{drop}}(i)$ and *l*_*m*_. ${P}_m^{\mathrm{drop}}(i)$ is the probability that packet *m* would be dropped by node *i* according to its cooperative willingness and ${P}_m^{\mathrm{drop}}(i)=1-{W}_m^{\mathrm{com}}(i)$. It is obvious that packet *m* is not very worth keeping when ${P}_m^{\mathrm{drop}}(i)$ is high. *l*_*m*_ is the proportion of packet *m*’s residual lifetime to packet time to live (TTL). The worthiness of keeping packet *m* declines with the decrease of *l*_*m*_, because the probability that the packet would be delivered within the residual lifetime becomes low when the deadline is close. Based on the above four factors, we define a four-dimensional space to describe the worthiness of keeping a packet and let packet profile vector ${V}_m=\left({D}_m(i),{D}_m\left(\varOmega \right),{P}_m^{\mathrm{drop}}(i),{l}_m\right)$ be a point of indicating packet *m*’s profile in the space. We use the benchmark vector *V*_*b*_ = (1,0,0,1) to indicate the situation when packet *b* is fully worth keeping, i.e., when the delivery probability of the carrier for packet *b* is 1, the delivery probability of others for this packet is 0, the dropping probability is 0, and packet *b* is newly generated with the maximum lifetime. Then the worthiness of carrying packet *m* is indicated by the similarity between packet *m* and packet *b*. To measure the similarity of the two packets, we use the weighted Euclidean distance given by Eq. (10), where ${v}_{m_{{}_k}}$ and ${v}_{b_k}$ are the *k*th elements of packet *m*’s and packet *b*’s profile vectors, respectively, and *σ*_*k*_ is used to adjust the relative importance of the *k*th element. The shorter the distance, the more similar the two packets are and the more packet *m* is worth keeping.

10 $$ {S}_{m,b}=1/\sqrt{{\displaystyle \sum_{k=1}^4{\sigma}_k{\left({v}_{m_{{}_{{}_k}}}-{v}_{b_{{}_{{}_k}}}\right)}^2}} $$

Let *Q*_*m*_(*i*) denote the *quality* of packet *m* on node *i* and *Q*_*m*_(*i*) = *S*_*m*,*b*_. When the buffer is not sufficient, packets with high *qualitie*s are more worth keeping by the carrier than those with low *qualitie*s.

#### Buffer allocation

Based on their importance, packets on a node are classified into *prime* packets and *ordinary* packets. Packet *m* is associated with ${d}_{\mathrm{m}}^{\max }$ which indicates the highest delivery probability that it has ever seen. If ${d}_m^{\max } = {D}_m(i)$, i.e., node *i* has the highest delivery probability that packet *m* has ever seen, then packet *m* is a *prime* packet for node *i*; otherwise, it is an *ordinary* packet.

**Fig. 2:**
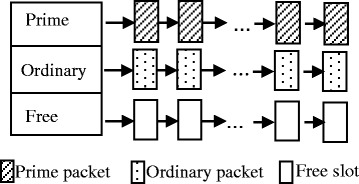
Organization of buffer

The buffer of a node is divided into many slots with fixed sizes. For simplicity, we assume all packets have the same size and each packet occupies a slot. When packets have variable sizes, it is easy to extend this mechanism by allocating different numbers of slots to packets with different sizes. As shown in Fig. 2, the slots are organized in three different queues named *prime* queue, *ordinary* queue, and *free* queue. The prime packet with the smallest quality is put at the tail of the prime queue. Similarly, the ordinary packet with the smallest quality is put at the tail of the ordinary queue. The free queue is composed of empty slots.

When a node receives or generates a new packet *m*, it allocates a free slot to this packet from the free queue. When there is no free slot, if packet *m* is a prime packet or an ordinary packet with a quality larger than the packet at the tail of the ordinary queue, it overwrites the latter; otherwise, it is dropped. There may be the case that the buffer is full but there are only prime packets in the buffer. In this case, if packet *m* is a prime packet and has a higher q*uality* than the one at the tail of the prime queue, it overwrites the latter; otherwise, it is dropped. Packet *m* is put into the appropriate queue after being accepted. When the node deletes packet *m*, the slot occupied by packet *m* is released and put into the free queue. Note that the node does not have to run the sort algorithm whenever a new packet arrives, it only need to run the algorithm when there is no free slot to allocate and the packet at the tail of the relevant queue is not with the smallest quality. Moreover, in this case, it only needs to run the bubble sort algorithm for the first loop to pick up the packet with the smallest quality to overwrite it. Thus, the time complexity is *O*(*n*) and the space complexity is *O*(1), where *n* is the number of packets in the relevant queue.

With the above scheme, prime packets have higher priorities than ordinary packets. Nodes keep prime packets longer than ordinary packets and keep packets with high *qualities* longer than those with low *qualities* when the buffer space is not sufficient. This mechanism can exploit the buffer more efficiently since packets that are more worth keeping are dropped with a lower probability.

### Forwarding decision

#### Forwarding pattern switch

**Fig. 3:**
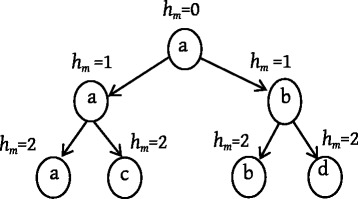
Number of duplication hops growing in the replication pattern

Packets are forwarded in the replication pattern at first after they are generated. Each packet is associated with the number of *duplication hops* to indicate the number of hops that the packet has been replicated. The number of *duplication hops* of packet *m* is denoted by *h*_*m*_. As shown by the example in Fig. 3, *h*_*m*_ is 0 when packet *m* is newly generated by node *a*, and it grows when the replication happens. A node keeps the copy of packet *m* and updates *h*_*m*_ after replicating it to the next hop.

To reduce the transmission cost, when the number of *duplication hops* reaches the *duplication threshold* (*DT*), the packet transmission pattern switches from the replication pattern to an entrusting pattern. In the entrusting pattern, a node will delete the copy of a packet after forwarding it to the next hop.

#### Forwarding algorithm

When a connection opportunity occurs, it is important to forward packets that are more worth forwarding since the connection between nodes is not permanent and the resources may not be sufficient. Suppose node *i* meets node *j*. If node *j* is the collection point, node *i* delivers all packets to the collection point and the forwarding completes. Otherwise, node *i* and node *j* exchange the delivery probabilities, and node *i* determines a candidate list *L* of packets. That is, for any packet *p* node *i* carries, if *D*_*p*_(*i*) < *D*_*p*_(*j*), node *i* puts packet *p* into *L*. After *L* is complete, node *i* forwards packets to node *j* following the steps below.

- First, for each packet in *L*, node *i* determines its type and quality on node *j*.
- Second, node *i* sorts *L* based on the packets’ types and qualities on node *j*, that is, prime packets are in front of ordinary packets, and packets with high *qualities* are in front of those with low *qualities* for each type. This is to ensure packets are forwarded in a decreasing order of their worthiness.
- Third, node *i* sends packets in *L* from head to tail to node *j*. For each packet *m* in *L*, if node *i* receives a feedback of *ACK*, which indicates packet *m* has been received by node *j*, node *i* checks whether the number of *duplication hops* exceeds the *duplication threshold*. If *h*_*m*_ < DT, it is in the replication pattern. In this case, node *i* updates *h*_*m*_ and updates ${d}_m^{\max }$ to *D*_*m*_(*j*) if ${d}_m^{\max}\le {D}_m(j)$. If *h*_*m*_ = DT, it is in the entrusting pattern and node *i* deletes packet *m* after it has been received by node *j*. If node *i* receives a feedback of *REJ*, which indicates packet *m* is rejected by node *j* due to buffer overflow, node *i* stops forwarding the rest of the packets in *L*.

**Fig. 4:**
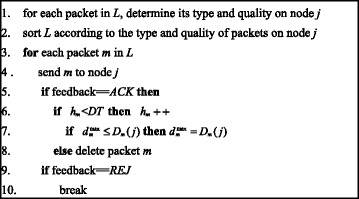
Pseudo-code of forwarding algorithm

The details of the forwarding algorithm are illustrated in Fig. 4. The time complexity of the algorithm is *O*(*l*log*l*) and the space complexity is *O*(*l*) in the worst case (i.e., when all packets in *L* are prime packets or ordinary packets, the time complexity is *O*(*l*log*l*) and the space complexity is *O*(*l*) when the merge sort method is used), where *l* is the number of packets in *L*. This is acceptable because most handsets have such computing capabilities.

It is worth noticing that node *i* sorts and forwards packets in *L* in decreasing order of their types and *qualities* from node *j*’s point of view. This forwarding decision has the advantage that packets forwarded to node *j* will not be deleted easily by node *j*. Moreover, when receiving a *REJ* message which indicates packet *m* is rejected by node *j* due to buffer overflow, node *i* does not need to forward the rest of the packets in *L* to node *j*, because compared to packet *m*, the rest of the packets in *L* have lower priorities or lower *qualities* on node *j*, and they will be also rejected by node *j*. This mechanism can avoid needless transmission and consequently can reduce the transmission cost.

Here we only describe the forwarding decision of node *i*. Packets forwarding from node *j* to node *i* take place in similar ways.

## Simulations

### Simulation setup

We evaluated the performance of ANT over two traces, MIT Reality [26] and Haggel Inforcom06 [27]. In the first trace, 97 smart phones were deployed to students and staff at MIT over a period of 9 months. These phones were running software that logged contacts with other Bluetooth-enabled devices. The second trace, which is referred to as Inf06 in this paper features 98 nodes; 78 of them are iMotes carried by conference participants, and the remaining 20 are fixed nodes situated at various places in the conference hotel such as conference rooms, the bar, the concierge, and the hotel elevators. Table 1 summarizes their main characteristics.

**Table 1:** Characters of the two traces

Experimental data set	Reality	Inf06
Device	Phone	iMote
Network type	Bluetooth	Bluetooth
Duration (days)	246	3
Granularity (seconds)	300	120
Number of contacts	110,000	191,000

As buffer space and power are the most important resources for mobile phones, we take both items into consideration in deciding the resource factor and allocate the same weights to them. To initialize the reciprocity level at the beginning, the number of packets that nodes have relayed for each other is initialized randomly between [0, 50]. The collection point drops the duplicate packets from one source if it receives them. We compare ANT against SSAR [21] and Bubble [11]. Since Bubble does not consider nodes’ selfishness and the buffer management, for a fair comparison, we modify it such that nodes work based on the same notion as G2G [20], i.e., they are cooperative with nodes from the same community and selfish with outside nodes, and packets are transmitted in decreasing order of the residual lifetime, because a packet with a long lifetime would be more likely delivered than a packet with a short lifetime before being dropped due to expiration. We also apply three packet-dropping policies (drop the tail, drop randomly, and drop the oldest) for Bubble in simulation and only present the results of the best policy here, i.e., drop the oldest (as the probability that the oldest packet would be delivered before expiration is relatively low compared to those with long residual lifetimes). We believe such refinement does not favor ANT in comparison. The modified Bubble is called Bubble_M. The collection points are regarded as normal nodes in Bubble_M to complete the community construction.

In each run, we use the first one third (1/10) of the Reality (Inf06) trace as the warm-up stage. Data collection is carried out in the remaining part. The packet generation of each node follows a Poisson process with an average arrival interval of 1 h (10 min) for the Reality (Inf06) trace. To avoid end effects, no packet is generated in the last TTL. The parameters and their default values are summarized in Table 2. We are interested in the following metrics for performance evaluation.

**Table 2:** Default values of parameters

Parameter	Value
Number of nodes	97 (Reality), 78 (Inf06)
Packet size (KB)	50
Deadline	10 days (Reality), 48 h (Inf06)
Buffer size (MB)	12
Bandwidth (Mbps)	2
*T* (hour), *α*, *H*	1, 0.8, 10
*σ*_1_, *σ*_2_, *σ*_3_, *σ*_4_	0.25
*w*_1_ (for buffer), *w*_2_ (for power)	0.5, 0.5
Battery capacity	1500 mAh
Threshold for the resource	40–80 %
DT	3
Number of collection points	2

#### Delivery ratio

The proportion of packets that have been delivered out of the total unique packets created within the deadline.

#### Delivery cost

The total number of packets transmitted across the air. To normalize this, we divide it by the total number of unique packets created.

#### Delivery delay

It is the duration from the time a packet is generated to the time the packet is delivered. Average delay for all packets is used for this metric.

### Results

#### Impact of deadline and number of collection points

**Fig. 5:**
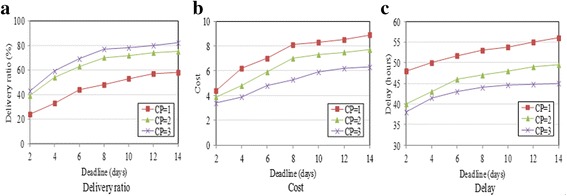
Impact of deadline and number of collection points (Reality). **a** Delivery ratio. **b** Cost. **c** Delay

Figure 5 presents the performance of ANT using the Reality trace by varying the packet deadline. It also presents the performance under different numbers of collection points. Packets that cannot be delivered within the deadline are dropped. We can see from Fig. 5a that the delivery ratio increases when the deadline extends, as packets can stay longer in the network and more packets can be delivered within the deadline. For that reason, the delivery cost and the average delay also increase, as shown in Fig. 5b, c.

It is also shown in Fig. 5 that the network performance improves with the increase of the number of collection points. Figure 5a shows that the delivery ratio increases with the existence of more collection points, because the packets exhibit a better opportunity to reach the collection points with more collection points existing. Meantime, with more collection points existing, the delivery cost decreases and the delay declines, as shown in Fig. 5b, c, respectively, because with more collection points existing, the packet can be delivered with fewer hops, thus reducing the energy consumption and shortening the delivery delay.

**Fig. 6:**
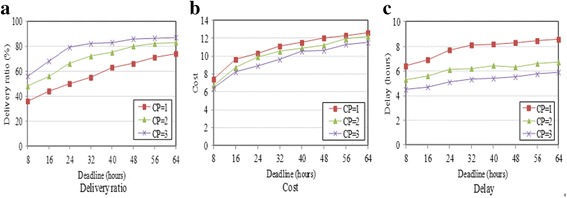
Impact of deadline and number of collection points (Inf06). **a** Delivery ratio. **b** Cost. **c** Delay

Similar results are shown in Fig. 6 using the Inf06 trace.

#### Impact of selfishness feature

**Fig. 7:**
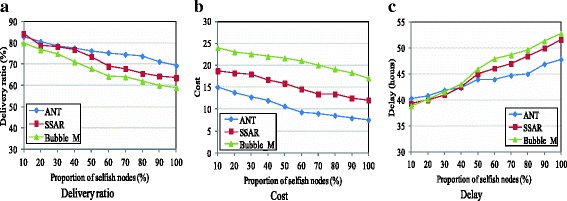
Impact of selfishness (Reality). **a** Delivery ratio. **b** Cost. **c** Delay

Figure 7 presents the impact of nodes’ selfishness on the performance and compares the protocols using the Reality trace.

As shown in Fig. 7a, the delivery ratio of all protocols decreases with an increase of the proportion of selfish nodes, because forwarding opportunities become fewer when nodes behave selfishly. For that reason, the delivery cost declines and the delay becomes longer, as shown in Fig. 7b, c, respectively. When most nodes are cooperative, the delivery ratio and delay of all protocols are similar. However, ANT achieves this performance at a much lower delivery cost than SSAR and Bubble_M. ANT performs better when more and more nodes behave selfishly. When over 40 % nodes behave selfishly, ANT outperforms SSAR and Bubble_M and achieves the best delivery ratio with the lowest cost and the shortest delay. This is due to the fact that ANT incorporates the contact probability and the cooperative willingness of nodes to evaluate the delivery abilities of nodes and it takes the status of the resources into account in relay selection. Moreover, it considers the worthiness of forwarding a packet based on an efficient buffer management scheme. Thus, it can exploit the forwarding opportunities and the buffer space more efficiently, which has positive effects on its delivery performance. However, SSAR and Bubble_M ignore the impact of resource constraint on the cooperative willingness; thus, packets would be dropped easily by the relays if they are not willing to carry and forward them due to the resource constraints. Moreover, they lack evaluations of the worthiness of forwarding a packet, and the transmission opportunities cannot be exploited efficiently. In addition, to solve the NP-hard MKPAR problem in forwarding decision, SSAR uses a greedy algorithm in substitution, which is simple but may be far from the optimum.

#### Impact of resource

As the cooperative willingness of selfish nodes is affected by their resource constraint, in this section, we compare the performance of proposed protocols under different battery capacities and buffer sizes.

**Fig. 8:**
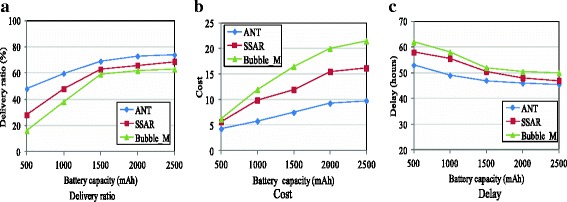
Impact of power (Reality). **a** Delivery ratio. **b** Cost. **c** Delay

The results of the algorithms over the Reality trace are shown in Fig. 8. The battery capacities are varied from 500 to 2500 mAh. Since we are concerned with the impact of the energy status on the delivery performance rather than the energy consumption process, we simply assume the lifecycle of the battery ranging randomly from 24 to 120 h for different users as different users may use the their phones in different ways. A new lifecycle restarts when the power is depleted. Figure 8a shows that the delivery ratio increases with the increase of the battery capacity, because the nodes’ cooperative willingness improves with the increase of the residual energy. For that reason, the delivery cost also increases and the delivery delay declines, as shown in Fig. 8b, c, respectively. ANT performs better than the other two protocols. It achieves a higher delivery ratio at a lower delivery cost and delay, especially when the battery capacity is low. The reason is that SSAR and Bubble_M do not consider the impact of the energy constraint on the cooperative willingness of nodes in relay selection and packets are very likely dropped when they are forwarded to nodes with low energy. ANT can mitigate the impact of this problem because the resource factor is taken into account in the relay selection.

**Fig. 9:**
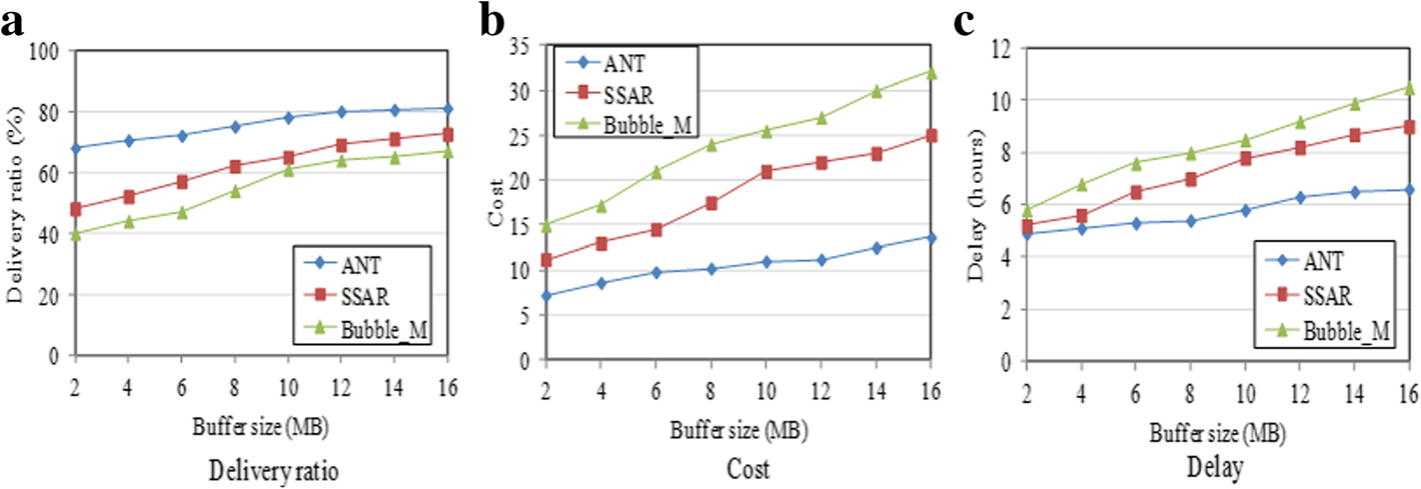
Impact of buffer size (Inf06). **a** Delivery ratio. **b** Cost. **c** Delay

Figure 9 shows the impact of the buffer size on the performance of the three protocols over the Inf06 trace. With the increment of the buffer size, the delivery ratio of all protocols increases and the cost and delay also increase. The reasons are that for one thing the cooperative willingness of nodes improves with the increase of the available buffer size and for another that packets can stay longer in the network before the buffer overflows. ANT performs better than SSAR and Bubble_M with the increase of the buffer space. For instance, it outperforms SSAR and Bubble_M by 40 and 70 %, respectively, in delivery ratio when the buffer size is 2 MB. It also achieves the lowest cost and the shortest delay. One reason is that ANT considers the buffer space constraint in deciding nodes’ forwarding willingness and the forwarding willingness is considered in relay selection. Another reason is that ANT uses an efficient buffer management scheme based on the type and quality of packets, and thus, it can exploit the forwarding chance more efficiently.

## Conclusions

In this paper, we proposed a routing protocol (ANT) tailored to data collection in a mobile crowdsensing environment. ANT works by accepting and tolerating the social selfishness of nodes. It integrates the contact opportunities and the cooperative willingness of nodes to make relay selections. It also incorporates a scheme of assessing the worthiness of carrying and forwarding a packet and employs that in buffer management and forwarding decisions. Extensive simulations using the MIT Reality trace and the Infocom06 trace demonstrate that ANT can exploit the forwarding chances effectively and it outperforms two other comparable protocols when nodes behave selfishly.
